# Tobacco Use Behaviors and Associated Factors among Newly Diagnosed Tuberculosis Patients in Benin and Burkina Faso

**DOI:** 10.3390/tropicalmed9060120

**Published:** 2024-05-22

**Authors:** Abdoul R. Ouédraogo, Arnauld A. Fiogbé, Sonia Menon, Marius Atchéni Esse, Tandaogo Saouadogo, Adam Daouda, Adjima Combary, Gildas Agodokpessi, Georges Ouédraogo, Gisèle Badoum, Yan Lin, Kobto G. Koura

**Affiliations:** 1 Service de Pneumologie, Centre Hospitalier et Universitaire de Tengandogo (CHU-T), Ouagadougou P.O. Box 104, Burkina Faso; 2 Unité de Formation et de Recherche en Sciences de la Santé, Université Joseph Ki-Zerbo, Ouagadougou P.O. Box 7021, Burkina Faso; georges.ouedraogo@gmail.com (G.O.); gisele.badoum.consultant@theunion.org (G.B.); 3 International Union Against Tuberculosis and Lung Disease, 75001 Paris, France; arnauld.fiogbe.consultant@theunion.org (A.A.F.); sonia.menon.consultant@theunion.org (S.M.); daoudaadam@gmail.com (A.D.); ylin.consultant@theunion.org (Y.L.); 4 National Tuberculosis Programme, Cotonou P.O. Box 321, Benin; essemarius.me@gmail.com (M.A.E.); aggildas2@gmail.com (G.A.); 5 Service de Pneumologie, Centre National et Universitaire de Pneumo-Phitsiologie de Cotonou (CNHUPP/C), Cotonou P.O. Box 321, Benin; 6 Epitech Research, 1160 Auderghem, Belgium; 7 National Tuberculosis Programme, Ouagadougou P.O. Box 6632, Burkina Faso; 8 Service de Pneumologie, Centre Hospitalier et Universitaire de Yalgado Ouédraogo (CNHU-YO), Ouagadougou P.O. Box 7022, Burkina Faso; 9 COMUE Sorbonne Paris Cité, Faculté des Sciences Pharmaceutiques et Biologiques, Université Paris Descartes, 75006 Paris, France

**Keywords:** tuberculosis, tobacco use, Benin, Burkina Faso

## Abstract

The objective of this study was to assess tobacco use (TU) behaviors among newly diagnosed pulmonary TB (PTB) patients and identify associated factors in Benin and Burkina Faso. A cross-sectional study was conducted in 20 randomly selected TB clinics. To ensure a representative study cohort, clinics were stratified during the sampling process. PTB patients were consecutively sampled in 20 of the clinics between 1 December 2021 and 30 September 2022. The study population comprised individuals aged 15 years and above who were newly diagnosed with PTB. Among the 1399 registered PTB patients, 564 (40.3%) reported a history of TU, including 392 (28.0%) current tobacco users and 172 ex-tobacco users. Cigarettes emerged as the predominant form of TU (86.2%), followed by smokeless tobacco (6.4%), and chicha smoking (2.6%). Factors independently associated with tobacco use were male gender (*p* < 0.001), being in Burkina Faso (*p* < 0.001), and an age of 25–59 years (*p* = 0.002). Our multicentric study reveals a substantial prevalence of tobacco use among TB patients, with cigarette smoking emerging as the predominant form. These findings underscore the imperative for implementing targeted cessation interventions within TB control programs. Special emphasis is warranted for male patients aged 25–59 years.

## 1. Introduction

Around 8 million people die from a tobacco-related disease every year, including an estimated 1.3 million non-smokers who are exposed to second-hand smoke, with over 80% deaths occurring in low- and middle-income countries [1,2,3]. Although the prevalence of tobacco use (TU) in Africa has generally been lower compared to other regions, the current TU rate in certain age groups surpasses that of other regions. The marketing of tobacco products, coupled with future projections indicating rapid increases in smoking, poses a double challenge for the region’s disease burden. Available data underscore the highly heterogeneous prevalence of adult TU across African countries, with rates ranging from 7.49% in Benin, 13.6% in Burkina Faso, to 37.6% in Sierra Leone [4,5].

Worldwide, tuberculosis (TB) remains a major global public health problem and is one of the leading causes of death [6]. Previous studies have shown that smoking is an independently confirmed risk factor for TB infection and progression from TB infection to active TB disease, increasing the risk of recurrence after successful TB treatment, delays in accessing health services, and having unfavorable treatment outcome [7,8,9].

Currently, more than 20% of global TB incidence may be attributable to smoking [7]. This may be attributed to smoking-induced damage to the lungs and immune system, which heightens susceptibility to both latent [10] and active TB [11]. Such damage alters immune responses and impacts immune cells such as macrophages, monocytes, and CD4 lymphocytes [12]. Additionally, a recent meta-analysis reported that the odds of TB recurrence among smokers were estimated to be 2.1 times higher than those of the non-smokers [13]. Also, smoking may lead to increased aerosolization of TB bacilli by damaging the lungs and respiratory tract, making it easier for the bacteria to be expelled into the air [14]. Furthermore, smokers with TB often experience poorer treatment outcomes, including higher rates of treatment failure and drug resistance [9].

In 2007, the World Health Organization (WHO) and International Union Against Tuberculosis and Lung Disease (the Union) advocated for the inclusion of smoking cessation within TB programs [15].

Whilst there is ample evidence demonstrating the feasibility and effectiveness of smoking cessation intervention [16], it has yet to be meaningfully incorporated into national TB programs in most high TB burden countries, with a critical starting point being the identification of tobacco users among TB patients. To date, scarce data exist on TU among TB patients in sub-Saharan African countries, especially in Francophone Africa. We therefore conducted operational research on the feasibility of the integration of smoking cessation intervention into routine TB service in Benin and Burkina Faso. As part of this study, the aims are to (1) describe type and pattern of TU among newly registered TB patients; (2) identify factors associated with TU.

## 2. Materials and Methods

### 2.1. Study Approach

This multicentric cross-sectional study was nested within an observational cohort prospective study. In this observational cohort prospective study, one group received routine care, while the second group was exposed to a smoke-free environment, health provider training, and the ABC smoking cessation strategy (A = Ask, B = Brief Advice, and C = Cessation Support) [15]. The observational prospective cohort study was conducted across 20 health centers in Benin and Burkina Faso. It enrolled individuals aged ≥15 newly diagnosed with pulmonary tuberculosis (PTB) between 1 December 2021 and 30 September 2022. This cross-sectional study utilized all data collected upon inclusion for both groups.

The study, conducted within the framework of Benin and Burkina Faso TB Programs, randomly selected ten TB clinics in each country. These countries were selected based on their TB burden, significant public health concerns related to smoking (with prevalence rates of current smoking recorded at 3.85% in Benin in the WHO STEPS 2015 survey and 9.6% in Burkina Faso in 2013) [17,18], advancements in TB control, and existing national policies prohibiting smoking in public places. To ensure a representative cohort for the study, a stratification sampling of clinics was conducted by economic development levels, security conditions, and diverse types of PTB cases. The selection process also considered the willingness of staff to participate in the study without imposing additional financial requirements.

### 2.2. Study Site and Population

Inclusion criteria for TB centers comprised the capacity to adhere to national TB treatment guidelines, a demonstrated willingness to participate, an annual registration of at least 50 TB patients, and the ability to implement the intervention without additional resources. Patient inclusion criteria encompassed newly diagnosed pulmonary bacteriologically confirmed TB (PTB+) cases aged 15 or older, with a stable residence in the region for the next 2–3 years, drug-sensitive or unknown status, and a demonstrated willingness to participate, providing informed consent for a 2–3 years follow-up. Exclusion criteria encompassed those unable or unwilling to implement the intervention, individuals already 100% tobacco-free with routine cessation activities, patients undergoing current TB treatment, those with confirmed drug-resistant TB (ineligible for first-line treatment), and individuals experiencing severe mental illness or disabling comorbidities.

### 2.3. Sample Size Determination and Sampling Strategy

A sample size for the observational prospective cohort study was estimated using the Schwartz formula: $n\geq \frac{{\;Z}_{1-\frac{\alpha }{2}}^{2}p\left(1-p\right)}{e^{2}}$, where *p* is anticipated prevalence of TU in the population, $Z_{1-\alpha /2}$ is the percentage of the standard distribution corresponding to the two-sided significance level (for the significance level of 5%, $Z_{1-\alpha /2}$ = 1.96), and e = 0.05 is the level of precision. A 10% non-response rate gives a total sample size of *N* = 424.

We utilized a stratified sampling approach across 102 TB clinics in Burkina Faso and 99 in Benin. Each TB clinic had an average capacity of 50, managing 25 PTB+ cases per semester. Twenty TB clinics with a notification of at least 50 PTB+ cases in 2019 were identified and proportionally allocated a sample size based on the 2019 PTB+ patient count in each clinic. However, due to security reasons in Burkina Faso, convenience sampling for the 20 eligible clinics was employed. Sequential inclusion of TB patients diagnosed with GeneXpert continued at each site until the predetermined sample size was achieved. Following registration, we collected comprehensive data, including residential addresses, telephone contacts, smoking statuses, and initial demographic and clinical characteristics.

### 2.4. Data Collection and Analysis

We established distinct subcategories within our variables for the primary outcome of interest, namely TU. A non-tobacco user was defined as an individual who had neither consumed a minimum of 20 packs of cigarettes in their lifetime nor smoked one cigarette per day for at least one year [16]; an ex-tobacco user as someone who had previously used tobacco but abstained for over 90 days before TB registration; and a current tobacco user as an individual who had consumed a minimum of 20 packs of cigarettes or an equivalent amount of other types of tobacco in their lifetime, or one cigarette per day for a duration of 30 days or more and was actively engaging in daily smoking at the time of registration [16]. The word “equivalent” refers to other forms of cigarette than industrial (e.g., hand-rolled cigarettes).

Patient data were entered into a designed digital platform. Data were analysed with Stata v18.0 (StataCorp, College Station, TX, USA). Student’s *t* test, chi square test (or Fisher’s exact test for small numbers), with 5% significance level (*p* < 0.05) was used to compare means in independent data/groups. Variables with *p* < 0.25 in univariate analysis were included in multivariate analysis. A stepwise logistic regression model guided by Akaike information criterion selected the final model variables. Variables’ impact on the model was evaluated using the likelihood ratio test. Adjusted odds ratios (aOR) with accompanying 95% confidence intervals (CIs) were calculated. The final model’s fit was examined using Hosmer and Lemeshow’s C statistic.

### 2.5. Ethics

The study was approved by The Health Ethics and Research Committee (CERS) of Burkina Faso (Approval Code: 2021-08-195, Approval date: 9 August 2021) and The Local Biomedical Research Ethics Committee of the University of Parakou (CLERB-UP) (Approval Code: 0465/CLERB-UP/P/SP/R/SA, Approval date: 9 August 2021).

## 3. Results

### 3.1. Characteristics of the Study Population

There were 1399 patients with PTB+ consecutively registered in this study, including 1076 (76.9%) male and 323 (23.1%) female. Their age range was 15–90 years with a median age of 37.0 years (IQR 27.5, 47.0). Most of the registered patients had no schooling or at most a primary school level of education (67.8%) (Table 1).

### 3.2. Characteristics of Tobacco Use

Out of the 1399 PTB+ patients, 564 (40.3%) had a history of using some form of tobacco product, with a higher percentage in Burkina Faso (49.6%; 95% CI: 45.9–53.3%) than in Benin (30.9%; 95% CI: 27.6–34.5%). Among current tobacco users among TB patients, the total was 393 (28%; 95% CI: 25.7–30.4), with a higher prevalence observed in Burkina Faso (33%) than Benin (23%). The prevalence of ex-tobacco users was 12.3% (95% CI: 10.6–14.1). TU was more frequent in male patients, the middle age group, and individuals with an employed position (Table 2).

The major type of TU was cigarette smoking, accounting for 86.2%, followed by smokeless tobacco (6.4%), and chicha smoking (2.6%). Among the registered TB patients who were currently smoking cigarettes (357), 24.1% of them reported smoking 20 or more cigarettes on a daily basis (Table 3).

There were 172 patients who used tobacco at some point but successfully quit before the commencement of this study (Table 4).

### 3.3. Factors Associated with Tobacco Use

The baseline characteristics of PTB+ patients with TU include demographic factors, education, employment status, and clinical disease type described in Table 1. Univariate analysis highlighted male gender, an age between 25 and 59 years, employment, and HIV-negative status as statistically significant factors associated with TU. In our final multivariate logistic regression model, male gender (*p* < 0.001), residence in Burkina Faso (*p* < 0.001), and belonging to the age group 25–59 years (*p* < 0.001) emerged as independent predictors of TU (Table 5).

## 4. Discussion

Our findings showed a high prevalence of TU among TB patients in Benin and Burkina Faso with cigarette smoking being the predominant form. Factors associated with TU were male sex, being in Burkina Faso, and an age of 25–59 years.

The primary form of TU identified in this study was cigarette smoking, aligning with findings reported in Benin and Burkina Faso [17,18]. The prevalence of current smokers among PTB+ patients in Burkina Faso and Benin surpasses the rates in their general populations by 3.4–6 times, respectively. In the context of sub-Saharan Africa, our study’s outcomes are analogous with findings from South Africa where 56% of PTB patients were identified as smokers, contrasting sharply with the 19.4% prevalence in the general population [19]. Additionally, similar trends were observed in Ethiopia, with 16.15% of TB patients reporting smoking habits, compared to 5.8% in the control group [20].

This study suggested a 6.4% prevalence of smokeless TU within the sampled population. When stratified by country, Benin reported a prevalence of 8.1%, in contrast to the 4.36% reported in Benin [18]. In Burkina Faso, the observed prevalence was 5.2%, lower than the 8.9% reported in 2013 (8.9%:(95% CI: 7.4–10.7) [17], although in Burkina Faso current use was defined as using smokeless tobacco on at least one day in the past 30 days.

Within this cohort, the TU behavior was alarming. Nearly 5% of current tobacco users simultaneously consumed several types of tobacco product or engaged in other forms of TU. It is noteworthy that 6 out of 10 patients identified as heavy smokers, reporting a daily consumption of 10 or more cigarettes. This trend was particularly pronounced in Burkina Faso, with 31.7% of heavy smokers engaging in daily smoking of at least 20 cigarettes. This high prevalence of heavy smoking suggests that cessation efforts may encounter additional challenges due to the psychological aspects of nicotine dependence. A retrospective study in Indonesia revealed that while most TB patients quit smoking during treatment, over one-third experienced relapses after treatment [21], highlighting the imperative for continuous monitoring of TU beyond treatment.

Among the 564 tobacco users, 12.3% had successfully quit tobacco entirely before their TB diagnosis. Regarding the duration of tobacco cessation leading up to the current TB diagnosis, nearly half of them had stopped tobacco consumption within the last 2 years, and over 80% had achieved this within a span of 9 years. This suggests an improvement in awareness of tobacco-related harms, possibly attributed to government health promotion strategies. However, the low prevalence of past smoking among the total registered TB patients who were tobacco users suggests that achieving cessation without cessation assistance may be challenging.

Our study identified independent predictors of TU, including residing in Burkina Faso. This finding prompts further investigation into factors such as people’s perceptions, awareness, socio-economic factors, or potential tobacco industry influence. Additionally, male sex, along with an age range of 25–59 presented an increased risk of TU use among PTB patients, aligning with trends reported in both countries and across SSA [4].

Our study brings attention to the substantial number of PTB patients with a history of TU, suggesting potential public health implications. This is because of the established link between smoking and adverse TB treatment outcomes coupled with an increased risk of post-treatment recurrence, emphasizing the need to identify tobacco users among PTB or presumptive PTB patients. This highlights the urgency of smoking cessation support within TB care programs.

The observation of a substantial proportion of smokers consuming more than 10 cigarettes in our study indirectly emphasizes the potential issues associated with second-hand smoke. By recognizing second-hand smoke as a risk factor for latent TB infection and progression to active TB disease, our study advocates for policies prohibiting smoking in public places. Furthermore, the identification of a subset of TB patients engaged in tobacco chewing adds another layer of concern, as tobacco chewing has been associated not only with health hazards such as gum disease, tooth decay, oral cancer [22], but also with poorer TB treatment outcomes [23].

Thus, monitoring TU beyond TB treatment is crucial, especially in regions like Burkina Faso, where heavy smoking is prevalent, and abstaining over an extended treatment course poses challenges. Our findings suggest the importance of tailored intervention strategies and cessation support programs within the context of TB care. Against a backdrop of a rapid increase in smoking projected in Sub-Saharan Africa [24] and a growing burden of non-communicable diseases (NCD) [25], our study underscores the necessity of targeted interventions, emphasizing comprehensive strategies for male TB patients in addressing the intersection of TB and TU.

### Strengths and Limitations

To our knowledge, this is the first multicentric investigation in West Africa to assess TU in TB patients within a routine programmatic setting, which enables real-world evidence data to be generated. Furthermore, the study benefited from a substantial number of patients consecutively enrolled through routine health services, minimizing the risk of selection bias. The inclusion of patients from both urban and rural/semi-urban areas enhanced the study’s representativeness, ensuring a diverse participant pool with varied TB types and smoking behaviors.

However, our findings should be seen within the context of certain limitations. The reliance on self-reported tobacco status, lacking confirmation through biochemical tests, introduces the potential for “social desirability bias”. In this scenario, some patients may not have accurately reported their TU behaviors. Furthermore, the convenience sampling of TB clinics in Burkina Faso, driven by geographic inaccessibility and security concerns, raises limitations on the study’s ability to fully represent the country’s true nature. However, as a compensatory factor, the study’s robust sample size and multicentric nature boost confidence. These strengths not only mitigate the potential impact of bias introduced by self-reporting but also enhance the overall validity of the study.

In 2018, Wang et al. undertook a systematic review and meta-analysis to quantitatively assess the association between drug-resistant TB and tobacco smoking. They included 33 studies and they found substantial evidence that tobacco smoking is associated with an increased risk of drug-resistant TB (OR 1.57, 95% CI 1.33–1.86) [26]. We did not include drug-resistant TB in the scope of our study (it was one of exclusion criteria). All TB patients in our study were drug-susceptible or had no evidence of drug-resistant TB. In these two countries, the total number of drug-resistant TB patients notified in 2021 was 35 in Benin and 83 in Burkina Faso [6] compared to the 1399 drug-susceptible TB patients included in our study. We did not have sufficient power to evaluate the relationship between drug-resistant TB and smoking status. Further studies are therefore necessary.

Our study emphasizes the importance of addressing epidemiological and operational gaps for optimizing tailored smoking cessation programs for TB patients. While providing quantitative insights into TU trends, our research underscores the significance of qualitative exploration in understanding cultural and social factors influencing smoking behaviors during TB treatment. This exploration may reveal intrinsic motivations and self-initiated strategies, essential for developing targeted support initiatives that address unique challenges in tobacco cessation for TB patients. With an anticipated rise in smoking rates, especially among men, along with the increasing burden of NCDs, a research gap exists in exploring comprehensive strategies at the intersection of TB and TU. This gap becomes even more crucial with emerging challenges such as diabetes [27], where the combined impact with TU has been shown to exacerbate the risk of mortality.

## 5. Conclusions

Our multicentric, cross-sectional study revealed a significant prevalence of tobacco use among TB patients, with cigarette smoking emerging as the prevailing form. Notably, these findings underscore the urgent need to integrate tailored cessation interventions into TB control programs, with a special emphasis on male patients aged 25–59 years.

## Figures and Tables

**Table 1:** Characteristics of the recruited TB patients in Benin and Burkina Faso (*n* = 1399).

Charasteristics	Country of Study		Total N (%)	*p*-Value
	Benin N (%)	Burkina Faso N (%)		
Gender				<0.001
Male	508 (73.1)	568 (80.7)	1076 (76.9)	
Female	187 (26.9)	136 (19.3)	323 (23.1)	
Age (year)				0.040
<25	131 (18.8)	103 (14.6)	234 (16.7)	
25–39	281 (40.4)	281 (39.9)	562 (40.2)	
40–59	233 (33.5)	246 (34.9)	479 (34.2)	
≥60	50 (7.3)	74 (10.6)	124 (8.9)	
Residence				<0.001
Urban	617 (88.8)	453 (64.3)	1070 (76.5)	
Rural	78 (11.2)	251 (35.7)	329 (23.5)	
Education				<0.001
No formal education	201 (28.9)	332 (47.2)	533 (38.1)	
Primary school or below	241 (34.7)	175 (24.9)	416 (29.7)	
High school or university	253 (36.4)	197 (27.9)	450 (32.2)	
Professional status				0.200
Employed	191 (85.0)	582 (82.7)	1173 (83.8)	
Unemployed	104 (15.0)	122 (17.3)	226 (16.2)	
Category of TB				0.120
New patient	643 (92.5)	635 (90.2)	1278 (91.4)	
Previously treated	52 (7.5)	69 (9.8)	121 (8.6)	
HIV status				0.060
Negative	625 (89.9)	653 (92.8)	1278 (91.4)	
Positive	70 (10.1)	51 (7.2)	121 (8.6)	
Tobacco using status				<0.001
Non-tobacco users	480 (69.1)	355 (50.4)	835 (59.7)	
Ex-tobacco users	55 (7.9)	117 (16.6)	172 (12.3)	
Current tobacco users	160 (23.0)	232 (33.0)	392 (28.0)	

**Table 2:** Characteristics of current tobacco users (*n* = 392).

	Number of Current Tobacco Users		Total N (%)	*p*-Value
	Benin N (%)	Burkina Faso N (%)		
Type of tobacco use				<0.001
Cigarette smoking	121(75.6)	217 (93.5)	338 (86.2)	
Smokeless tobacco	13 (8.1)	12 (5.2)	25 (6.4)	
Chicha smoking	8 (5.0)	2 (0.9)	10 (2.6)	
Others *	18 (11.3)	1 (0.4)	19 (4.8)	
Duration of tobacco use				0.053
<5 years	21 (13.1)	14 (6.0)	35 (8.9)	
5–9 years	24 (15.0)	27 (11.6)	51 (13.0)	
10–19 years	59 (36.9)	93 (40.1)	152 (38.8)	
≥20 years	56 (35.0)	98 (42.3)	154 (39.3)	
Gender				0.800
Male	156 (97.5)	224 (96.6)	380 (96.9)	
Female	4 (2.5)	8 (3.4)	12 (3.1)	
Age				0.400
<25 years	18 (11.3)	17 (7.3)	35 (8.9)	
25–39 years	64 (40.0)	108 (46.6)	172 (43.9)	
40–59 years	69 (43.1)	92 (39.7)	161 (41.1)	
≥60 years	9 (5.6)	15 (6.4)	24 (6.1)	
Residence				<0.001
Urban	147 (91.9)	151 (65.1)	298 (76.0)	
Rural	13 (8.1)	81 (34.9)	94 (24.0)	
Education				0.013
No formal education	52 (32.5)	106 (45.7)	158 (40.3)	
Primary school or below	43 (26.9)	61 (26.3)	104 (26.5)	
High school or university	65 (40.6)	65 (28.0)	130 (33.2)	
Professional status				0.500
Employed	152 (95.0)	217 (93.5)	369 (94.1)	
Unemployed	8 (5.0)	15 (6.5)	23 (5.9)	
Category of TB				0.120
New patient	149 (93.1)	205 (88.4)	354 (90.3)	
Retreatment	11 (6.9)	27 (11.6)	38 (9.7)	

* Including TB patients who used more than one type of tobacco product.

**Table 3:** Smoking behavior of current smokers (cigarette smoking only) (*n* = 357).

	Number of Current Smokers		Total N (%)	*p*-Value
	Benin N (%)	Burkina Faso N (%)		
Daily consumption of Tobacco				<0.001
<5 cigarettes or equivalent *	44 (31.7)	18 (8.3)	62 (17.4)	
5–9 cigarettes or equivalent	44 (31.7)	40 (18.3)	84 (23.5)	
10–19 cigarettes or equivalent	34 (24.5)	91 (41.7)	125 (35.0)	
≥20 cigarettes or equivalent	17 (12.1)	69 (31.7)	86 (24.1)	
Total	139 (100.0)	218 (100.0)	357 (100.0)	

* The word “equivalent” refers to other forms of cigarette than industrial (e.g., hand-rolled cigarette).

**Table 4:** Duration (years) of quitting tobacco use for ex-tobacco users (*n* = 172).

	Number of Ex-Smokers		*p*-Value
Duration (Years) of Quitting Tobacco Use	Benin N (%)	Burkina Faso N (%)	
<2 years	21 (38.2)	54 (46.2)	0.500
2–9 years	20 (36.4)	43 (36.8)	
10–19 years	9 (16.4)	10 (8.5)	
≥20 years	5 (9.1)	10 (8.5)	
Total	55 (100)	117 (100)	

**Table 5:** Univariate and multivariate analysis of tobacco use in relation to patients’ characteristics (*n* = 1399).

	N = 1399	OR [95% CI]	*p*-Value	Adjusted OR [95% CI]	*p*-Value
Countries			<0.001		0.001
Benin	695	1		1	
Burkina Faso	704	1.64 (1.29–2.08)		1.51 (1.18–1.94)	
Residence			0.800		–
Urban	1070	1		–	
Semi-Urban	329	1.03 (0.78–1.35)		–	
Gender			<0.001		<0.001
Women	323	1		1	
Men	1076	14.14 (8.19–26.95)		12.6 (7.30–24.1)	
Age groups (years)			<0.001		<0.001
<25	234	1		1	
25–39	562	2.50 (1.69–3.79)		2.01 (1.33–3.10)	
40–59	479	2.87 (1.93–4.37)		2.17 (1.43–3.36)	
60+	124	1.36 (0.76–2.40)		1.09 (0.60–1.97)	
Education			0.300		–
High school and above	450	1		–	
Primary or middle school	416	0.82 (0.60–1.10)		–	
No formal education	533	1.03 (0.78–1.36)		–	
Professional status			<0.001		–
Employed	1173	1		–	
Unemployed	226	0.24 (0.15–0.37)		–	
Category of TB			0.400		–
New	1278	1		–	
Re-treatment	121	1.19 (0.79–1.77)		–	
HIV			0.007		–
Negative	1278	1		–	
Positive	121	0.51 (0.30–0.81)		–	

## Data Availability

The data that support the findings of the study are available from one of the first authors (K.G.K.) upon reasonable request.
